# Fibromuscular dysplasia

**DOI:** 10.1186/1750-1172-2-28

**Published:** 2007-06-07

**Authors:** Pierre-François Plouin, Jérôme Perdu, Agnès La Batide-Alanore, Pierre Boutouyrie, Anne-Paule Gimenez-Roqueplo, Xavier Jeunemaitre

**Affiliations:** 1 Hypertension unit and Centre National de Référence des Maladies Vasculaires Rares, Hôpital Européen Georges Pompidou, AP-HP, Université Paris Descartes, Faculté de Médecine, INSERM Unit 772, Collège de France, Paris, France; 2 Department of Genetics and Centre National de Référence des Maladies Vasculaires Rares, Hôpital Européen Georges Pompidou, AP-HP; Paris, France; 3 Department of Pharmacology, Hôpital Européen Georges Pompidou, AP-HP; Université Paris Descartes, Faculté de Médecine, INSERM Unit 337, Paris, France; 4 Department of Genetics and Centre National de Référence des Maladies Vasculaires Rares, Hôpital Européen Georges Pompidou, AP-HP, Université Paris Descartes, Faculté de Médecine, INSERM Unit 772, Collège de France, Paris, France

## Abstract

Fibromuscular dysplasia (FMD), formerly called fibromuscular fibroplasia, is a group of nonatherosclerotic, noninflammatory arterial diseases that most commonly involve the renal and carotid arteries. The prevalence of symptomatic renal artery FMD is about 4/1000 and the prevalence of cervicocranial FMD is probably half that. Histological classification discriminates three main subtypes, intimal, medial and perimedial, which may be associated in a single patient. Angiographic classification includes the multifocal type, with multiple stenoses and the 'string-of-beads' appearance that is related to medial FMD, and tubular and focal types, which are not clearly related to specific histological lesions. Renovascular hypertension is the most common manifestation of renal artery FMD. Multifocal stenoses with the 'string-of-beads' appearance are observed at angiography in more than 80% of cases, mostly in women aged between 30 and 50 years; they generally involve the middle and distal two-thirds of the main renal artery and in some case also renal artery branches. Cervicocranial FMD can be complicated by dissection with headache, Horner's syndrome or stroke, or can be associated with intracerebral aneurysms with a risk of subarachnoid or intracerebral hemorrhage. The etiology of FMD is unknown, although various hormonal and mechanical factors have been suggested. Subclinical lesions are found at arterial sites distant from the stenotic arteries, and this suggests that FMD is a systemic arterial disease. It appears to be familial in 10% of cases. Noninvasive diagnostic tests include, in increasing order of accuracy, ultrasonography, magnetic resonance angiography and computed tomography angiography. The gold standard for diagnosing FMD is catheter angiography, but this invasive procedure is only used for patients in whom it is clinically pertinent to proceed with revascularization during the same procedure. Differential diagnosis include atherosclerotic stenoses and stenoses associated with vascular Ehlers-Danlos and Williams' syndromes, and type 1 neurofibromatosis. Management of cases with renovascular hypertension includes antihypertensive therapy, percutaneous angioplasty of severe stenoses, and reconstructive surgery in cases with complex FMD that extends to segmental arteries. The therapeutic options for securing ruptured intracerebral aneurysms are microvascular neurosurgical clipping and endovascular coiling. Stenosis progression in renal artery FMD is slow and rarely leads to ischemic renal failure.

## Definition and diagnostic criteria

### Disease name/synonyms

Early descriptions of the disease used the terms fibromuscular hyperplasia or fibroplasia, but now the term fibromuscular dysplasia (FMD) is used. McCormack *et al *in 1958 reported a pathological description of fibromuscular hyperplasia in four patients with renovascular hypertension [1]. In 1965, Hunt *et al *[2] observed that the disease was heterogeneous and not necessarily associated with hyperplasia, and introduced the term FMD. It was soon recognized that FMD could be present in carotid arteries without documented renal artery FMD or hypertension [3]. FMD is currently defined as an idiopathic, segmental, non-inflammatory and non-atherosclerotic disease of the musculature of arterial walls, leading to stenosis of small and medium-sized arteries. FMD may be familial (OMIM #135580).

### Pathologic classification

A pathological classification of renal artery FMD was proposed by McCormack *et al *[4,5] and revised by Stanley [6]. It was based on the dominant arterial wall layer involved: the intima, media or adventitia. Three main types of FMD have been identified: intimal, medial and perimedial [6], and these types have also been described in extrarenal arteries [7]. Intimal FMD, which accounts for 5% or renal artery FMD cases, is characterized by irregularly arranged mesenchymal cells within a loose matrix of subendothelial connective tissue and a fragmented internal elastic lamina. Nearly 85% of all FMD stenoses in the renal arteries are medial FMD; the lesion is a homogeneous collar of elastic tissue that presents as multiple stenoses interspersed with aneurismal outpouchings, with a preserved internal elastic lamina. Perimedial FMD affects approximately 10% of dysplastic renal arteries and involves excessive tissue deposition at the junction of the media and adventitia. The three types are not mutually exclusive. Indeed, Stanley reported that medial and perimedial FMD lesions can coexist in the same arterial segment [6]; and Alimi *et al *analyzed arterial segments from 33 patients and found that more than one artery layer was involved in 25 (66%) of the 38 specimens studied [8].

Since reports of successful outcomes of angioplasty in patients with renal artery FMD [9], surgery has been used only rarely for patients with FMD and consequently histological verification is available for only a small minority of cases. As a result, contemporary reports infer the pathological type of FMD from the angiographic appearance of arterial lesions. Table 1 shows the distribution of pathological types and the angiographic appearance of FMD in two series in which both pathological and angiographic findings were reported [3,4].

**Table 1:** Pathological-angiographic correlations. Most common lesions in patients who underwent both angiographic and pathological renal artery examinations

	**Total**	**FMD type**	**Angiographic appearance**		
			
**Pathologic classification**	**Number**	**Distribution**	String of beads"	Focal	Tubular
**Mayo Clinic [3]**	60				
Intimal		5	0	2	3
Medial		53	38	3	12
Periarterial		2	0	0	2
**Cleveland Clinic [4]**	67				
Intimal		14	0	14	0
Medial		53	46	7	0
Periarterial		0			

### Pathological-angiographic correlations

**Figure 1:**
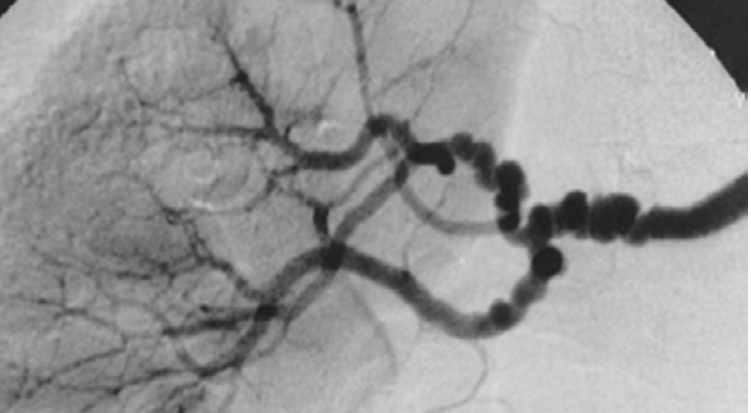
**The "string-of-beads" feature in medial fibromuscular dysplasia**. The sign is caused by areas of relative stenoses alternating with small aneurysms. The diameters of the aneurysms exceed the normal diameter of the artery. The sign is characteristic of medial FMD. A similar bead appearance may be seen in perimedial FMD, but the diameters of the beads do not exceed the normal diameter of the artery [10]. Note the involvement of branch renal arteries.

**Figure 2:**
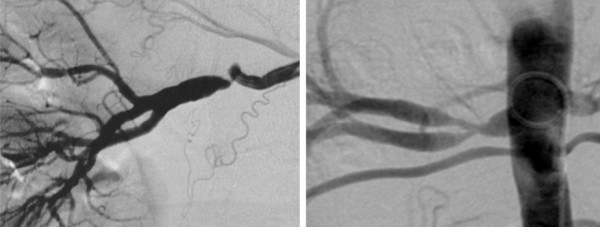
**Unifocal (left) and tubular (right) stenoses**. Unifocal stenoses are typically seen in intimal FMD. Tubular stenoses are not specifically associated with any one of the three dominant FMD types (intimal, medial or perimedial)

Kincaid *et al *described angiographic features in 125 patients with FMD, including 60 patients who underwent surgery and provided arterial tissue for histological examination [3]. They proposed an angiographic classification of FMD into four types. The multifocal type, with multiple stenoses and the 'string-of-beads' appearance, was present in 78 cases (62%); the tubular type, with a long concentric stenosis was present in 17 cases (14%); the focal type, with solitary stenosis less than 1 cm in length, was present in 9 cases (7%); and 21 patients (17%) had mixed-type stenoses. Among the 60 stenoses for which both radiological and histological assessments were available (Table 1), all 38 multifocal stenoses were associated with medial FMD, whereas focal and tubular stenoses were not specifically associated with histological type. Similar results were obtained in a pathological-angiographic correlation study at the Cleveland Clinic [4]. The "string-of-beads" sign (Figure 1) is the most suggestive and most frequent aspect of FMD [10], whereas focal and tubular stenoses only differ by the length of the stenosis with an arbitrary cut-off (Figure 2). A conservative conclusion is that multifocal stenoses with the "string-of-beads" feature is characteristic of FMD [3,4,10] and probably denotes the presence of the medial type FMD [3], whereas other angiographic aspects cannot be related to specific histological lesions.

### Arterial bed distribution

Most commonly affected are the renal and carotid arteries. Involvement of the axillary, iliac, basilar, hepatic and intracranial arteries has also been reported. In a comprehensive review of the literature, Mettinger and Ericson analyzed reports concerning a total of 1197 patients with FMD [11]. Renal arteries were involved in 58% of cases, cervicocranial arteries in 32%, and other arteries in 10%. The type of FMD and any coexistence of FMD in several arterial beds were not reported. In a series of 104 patients with renal artery FMD documented at angiography [12], 81 (78%) had the string-of-bead sign; FMD affected both renal arteries in 54 patients. Among the 81 patients in whom the carotid arteries were examined, 9 (11%) had carotid artery FMD. In a series of 37 patients with carotid artery FMD [13], 23 (62%) had the "string-of-beads" feature; half the patients were hypertensive, but none underwent renal artery angiography.

Unlike atherosclerotic stenoses, stenoses due to FMD rarely affect the ostial or proximal segments of arteries. FMD stenoses of renal arteries are usually truncal or distal and may involve arterial branches [3-7]; they are more frequent on the right side [3,7]. Most of carotid artery FMD lesions occur adjacent to the C1–2 interspace [13,14].

### FMD-related aneurysms and dissection

Macroaneurysms affecting the renal or carotid arteries are more frequent in FMD than in the general population (see Epidemiology section below). As intracranial aneurysms may rupture and lead to subarachnoid or intracerebral hemorrhage, the American Heart Association recommends performing magnetic resonance angiography of the head in patients with cervical artery FMD [15]. Spontaneous cervical artery dissections are a common cause of stroke in young and middle-aged adults and are associated with FMD in about 15% of cases [16]. Spontaneous renal artery dissections are rare but frequently coexist with FMD [17,18]. Aneurysms and dissections are considered to be complications of FMD but frequently arise in individuals with no FMD. Therefore, their presence without direct evidence of FMD does not suffice to diagnose the condition [6].

## Epidemiology

### Prevalence

The prevalence of FMD is not precisely known. Ten to 20% of documented renal artery stenoses are the consequence of FMD, and renovascular hypertension is documented in less than 1 to 2% of hypertensive patients. Assuming a 20% prevalence of hypertension in middle-aged subjects, the prevalence of clinically significant renal artery FMD can be estimated to be about 0.4%. The prevalence of asymptomatic renal artery FMD is, however, one order of magnitude higher: four reports of angiographic findings for a total of 3181 potential kidney donors describe 139 subjects (4.4%) with evidence of FMD [19-22]. The reports did not provide details concerning the radiological criteria used to define the condition, however. The number of reported cases of carotid artery FMD is about half that of renal artery FMD (see above) [11].

Renal artery aneurysms were identified in four of 716 (0.6%) potential kidney donors in one series, all four having lesions suggestive of FMD [21]; similarly 12 of the 125 (9.6%) patients with renal artery FMD reported by Kincaid *et al *had renal artery aneurysms [3]. The prevalence of intracranial aneurysms in the general population is estimated to be 1 to 5% [23] but was 7.3% in a meta-analysis combining data from 18 reports describing patients with carotid artery FMD [24]. The prevalence of carotid artery dissection in the general population is about 1 per 100,000. To our knowledge, there are no reliable estimates of the prevalence of renal artery dissection in the general population or of the prevalence of dissection in patients with cervical or renal artery FMD.

### Age and sex

**Figure 3:**
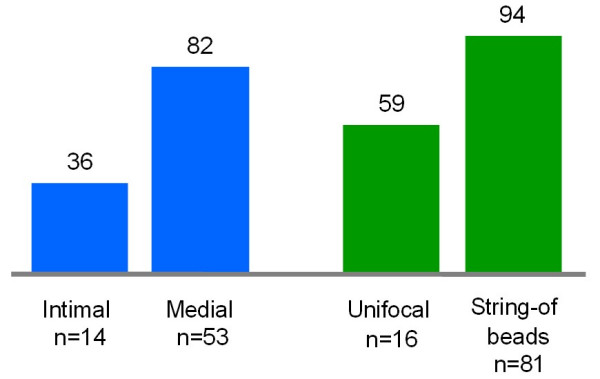
**Percentage of women by type of fibromuscular dyplasia**. The blue columns indicate the percentages of patients who are women according to a binary pathological classification (adapted from [4]), and the green columns the percentages of patients who are women according to a binary angiographical classification (adapted from [12]). Note that women make up the majority of cases of the most prevalent type, medial FMD, which is associated with the "string-of-beads" feature on angiography.

Medial-type FMD and/or FMD showing the "string-of-beads" appearance at angiography are usually diagnosed in young adults and are more than four times as frequent in women as in men. A male predominance in found in cases with intimal-type FMD or with focal stenoses at angiography, but these cases make up only a minority of patients with FMD [12] (Figure 3).

## Clinical description

### Clinical presentation in renal artery FMD

The most common clinical presentation of FMD is renovascular hypertension secondary to renal artery involvement. Renal artery stenosis due to FMD may be associated with all stages of hypertension, but it is most commonly detected in patients with stage 2–3 hypertension, or abrupt onset or resistant hypertension, since these are the individuals who undergo the most comprehensive etiological examinations. Upper abdominal quadrant or flank bruits are common in patients with renal artery FMD [6,12] but this clue has only limited diagnostic sensitivity and specificity [25]. FMD may be complicated by renal artery dissection and kidney infarction with abrupt flank pain, hematuria and rapidly progressive hypertension [18]. As a result of activation of the renin-angiotensin system, hypokalemia reflecting secondary hyperaldosteronism may be present, particularly in cases complicated with renal artery dissection and kidney infarction [18,26]. Unlike atherosclerotic renal artery stenosis, FMD renal artery disease is rarely associated with high serum creatinine levels.

### Clinical presentation in carotid artery FMD

Cervical artery FMD, mainly affecting the internal carotid artery, is seldom symptomatic. It can be discovered incidentally by Doppler ultrasound during evaluation of dizziness, headache, or an asymptomatic cervical bruit [11]. Neurological symptoms occur when FMD lesions are complicated by brain ischemia, embolus, and thrombosis in cases with dissection or associated aneurysms rupture. Complications may present as Horner's syndrome, transient ischemic attack, ischemic stroke or subarachnoid hemorrhage. The association in a given patient of hemorrhage due to aneurysm rupture and ischemic stroke due to stenosis is characteristic of cerebral FMD [13].

### FMD affecting extra-renal and extra-cervical sites

FMD affecting extra-renal and extra-cervical arteries has been reported in celiac, superior and inferior mesenteric, hepatic, splenic, and coronary arteries [7]. Although most patients with digestive artery FMD are asymptomatic, they can present with mesenteric ischemia (postprandial abdominal pain, weight loss and epigastric bruit), or rarely mesenteric infarction and multi-organ failure [27]. Patients with lower limb artery FMD may present with cold legs, intermittent claudication, or evidence of distal embolic disease. Patients with subclavian artery FMD may present with arm weakness, paresthesias, claudication, and subclavian steal syndrome [7,13].

## Etiology/genetics

Renal artery FMD has been tentatively associated with environmental factors and it is likely that there is a genetic predisposition.

### Environmental factors

More patients with FMD than matched controls smoke [7,28,29] and FMD patients who smoke have a more severe arterial disease than those who do not [30]. The mechanisms by which smoking contributes to the etiology of FMD have, however, not been elucidated. The well-documented majority of women among patients with FMD (Figure 3) suggests that exposure to endogenous or exogenous estrogens may predispose to the condition, but the number of pregnancies and the frequency of oral contraceptive use does not differ between patients with FMD and matched controls [7,29]. Renal mobility when assuming the upright position is greater in women than in men and in the right than in the left kidney [29]: FMD is more frequent in women than in men and in the right than in the left kidney, so it has been suggested that repeated stretching of the renal artery may cause micro traumas that predispose to FMD [7]. However Sang *et al *found that kidney mobility during maximal respiratory cycle or during a change in posture did not differ between patients with FMD and matched controls with normal renal arteries [29].

### Genetic factors

The occurrence of renal FMD in sib pairs or identical twins [31] suggests its possible inheritability. The first formal pedigree analysis was performed by Rushton [32] who suggested that FMD is transmitted as an autosomal dominant disease with incomplete penetrance and variable clinical symptoms. However, his conclusions were drawn from interviews of relatives who were considered to have FMD but whose arterial disease also included peripheral arterial occlusion or coronary heart disease, *i.e*. conditions more likely to be associated with atherosclerosis than with FMD. Our retrospective analysis of 104 patients with renal FMD showed a prevalence of 11% for familial cases, *i.e*. 11% of cases had at least one sibling with angiographic evidence of renal artery FMD [12]. However, familial detection based on angiography is not practicable in relatives who are normotensive or asymptomatic. As renal artery FMD may be associated with only mild hypertension or even present in normotensive subjects (see data concerning kidney donors above), the presence of FMD is probably overlooked in many relatives of index cases. High-resolution echo-tracking methods are promising alternatives to angiography for screening asymptomatic relatives because subclinical alterations of the common carotid artery wall seem common in patients with renal artery, medial FMD [33]. Using this technique, elevated echo-tracking scores of the carotid artery have been found in first-degree relatives of index cases and the findings are consistent with the possibility of autosomal dominant transmission [34].

Few molecular genetic studies have been conducted, because of the absence of large affected pedigrees and/or the absence of large and well-phenotyped cohorts allowing powerful case-control studies. Autoimmunity has been implicated by a limited study showing an association with HLA Drw6 [29] but this has not been confirmed. In a larger series involving 161 patients and 3 sets of controls, we did not find any association with functional polymorphisms of the **α**-1 antitrypsin gene [35]. Bofinger *et al *reported an association with polymorphisms of the renin angiotensin system [36] but this finding also has not been confirmed.

## Diagnostic methods

### Screening tests

**Figure 4:**
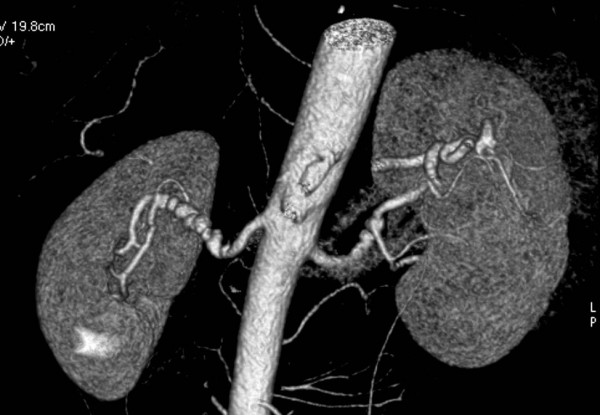
Computed tomography angiography in a patient with medial dibromuscular dysplasia.

The presence of renal artery FMD can be documented by the following non-invasive tests (in increasing order of accuracy): the captopril test, captopril renal scintigraphy, Doppler ultrasound, magnetic resonance angiography, gadolinium-enhanced magnetic resonance angiography, and computed tomographic angiography [37]. Doppler ultrasound is a time-consuming, examiner-dependent procedure and has several limitations including obesity, poor compliance for respiratory renal movement, and failure to visualize entire arteries or to define accessory arteries. Computed tomographic angiography is the most specific test in patients at risk of harboring FMD renal artery stenosis (Figure 4), but gadolinium-enhanced magnetic resonance angiography has the advantages of no radiation exposure and limited nephrotoxicity. A careful prospective multicenter comparative study found that computed tomographic angiography and gadolinium-enhanced magnetic resonance angiography had reasonably good specificities for detecting renal artery stenosis due to FMD (92 and 84 percent, respectively) but disappointing sensitivities (64 and 62 percent, respectively) [38].

There is no published comparative study of non-invasive tests for detecting carotid artery FMD, although Doppler ultrasound may disclose irregular patterns of stenosis that are suggestive. Computed tomographic angiography and magnetic resonance angiography are probably more effective than ultrasonography for detecting lesions of the middle and distal portions of the carotid and vertebral arteries and may also document or rule out the association of intracranial aneurysms.

### Confirmatory test

The commonly accepted gold standard for diagnosing renal artery FMD is intra-arterial angiogram with digital subtraction. This invasive test should be reserved for patients in whom it is clinically justified to proceed with revascularization in the same procedure. Stenosis quantification is, however, frequently difficult in medial FMD with the "string-of-beads" appearance because multiple web-like defects are often present in patients with FMD, contributing to clinically significant stenoses that may not be apparent on angiography.

## Differential diagnosis

The characteristic "string-of-beads" feature is found in the majority of patients with FMD (Table 1). In cases with focal and tubular FMD stenoses, differential diagnoses are atherosclerotic or inflammatory artery diseases, vascular Ehlers-Danlos and Williams' syndromes, and type 1 neurofibromatosis. FMD lesions are typically truncal or distal, as opposed to atherosclerotic stenoses that are mostly ostial or proximal; most patients with FMD are young and present few or no risk factors for atherosclerosis and no aortic plaques. In 178 consecutive angiograms from patients with FMD or atherosclerotic renal artery stenoses, readers blinded for age, sex, and clinical context generally agreed on the etiology (kappa coefficient 0.677) [39]. FMD differs from inflammatory diseases like Takayasu arteritis by the absence of inflammation or aortic stenosis. Patients with vascular Ehlers-Danlos syndrome, with the Williams syndrome, or with type 1 neurofibromatosis may have stenoses of renal and visceral arteries that mimic FMD. The diagnosis of these conditions relies on associated phenotypic traits and genetic tests: acrogeric dysmorphy, distal joint laxity and tiny skin elasticity in vascular Ehlers-Danlos syndrome (confirmed by detection of *COL3A1 *gene mutations) [40]; facial dysmorphy, supra-aortic stenosis and particular behavior in Williams syndrome (confirmed by detection of deletion 7q1.2 using FISH method) [41]; and specific skin lesions in neurofibromatosis 1 [42].

## Management

### Management of renal artery FMD

The value of treatment has not been established for renal artery FMD without hypertension. The management of hypertension associated with renal artery FMD involves revascularization and/or antihypertensive medication. There have been no controlled trials comparing revascularization to medication in FMD. Current recommendations reflect the knowledge acquired concerning atherosclerotic renovascular hypertension, although indications for balloon angioplasty are wider in FMD than in atherosclerotic renovascular disease because the blood pressure outcome of angioplasty is more favorable in FMD than in atherosclerosis [9]. Revascularization is recommended for patients with hemodynamically significant renal artery stenosis – *i.e*. with bilateral stenoses or a unilateral stenosis causing more than 60% reduction in luminal diameter – and accelerated hypertension, resistant hypertension, malignant hypertension, hypertension with an unexplained unilateral small kidney, and hypertension with intolerance to medication [15]. It is also useful in young patients with recent-onset hypertension and hemodynamically significant renal artery stenosis due to FMD: in these cases the goal is to cure the hypertension. The standard revascularization procedure is balloon angioplasty with bailout stent placement if necessary. Surgical reconstruction is indicated for patients with complex FMD that extends to segmental arteries and those with macroaneurysms [15]. Antihypertensive drug treatment is indicated for patients with long-standing hypertension or in cases with persistent hypertension following revascularization. Medication includes angiotensin-converting enzyme inhibitors, angiotensin receptor blockers, calcium channel blockers and betablockers. In cases with hemodynamically significant stenosis and no indication for primary revascularization, renal echography should be performed every year and revascularization considered if kidney length shortens by 1 cm or more.

### Management of symptomatic carotid artery FMD

Management of dissecting carotid artery FMD includes anticoagulation and, in cases with expanding or symptomatic pseudoanevrysm, percutaneous angioplasty or surgical repair [43]. Therapeutic options for securing ruptured intracerebral aneurysms are microvascular neurosurgical clipping and endovascular coiling [44].

## Natural history and prognosis

Renal artery FMD, mainly of focal or tubular types, may progress to more severe stenosis and rarely to renal artery occlusion [3,7,45-47]. Multivessel FMD may be rapidly progressive, particularly in young children, sometimes in a familial context [48,49]. Information regarding progression in FMD is limited and retrospective, however, and the risk of progression as assessed from available studies is probably overestimated because documentation of progression was obtained from intra-arterial angiography, a procedure which is not routinely undertaken in patients with favorable clinical and biological outcomes. Overall, progression of arterial lesions is considered less severe in FMD than in atherosclerotic renal artery stenoses [45,46]. We are not aware of any follow-up studies in patients with untreated cervicocranial FMD.

## Unresolved questions

The major aims of current research are to unravel the pathophysiological mechanisms of FMD; to seek gene(s) that predispose to the condition; to assess more accurately the risk of disease progression in focal or multifocal FMD, and in FMD affecting renal or extrarenal arteries; and to improve the detection and quantification of renal artery stenoses.

## Competing interests

The author(s) declare that they have no competing interests.

## Authors' contributions

All authors participated in the drafting and editing of this manuscript and have seen and approved the final version.
